# Does Measurement of Corticospinal Tract Involvement Add Value to Clinical Behavioral Biomarkers in Predicting Motor Recovery after Stroke?

**DOI:** 10.1155/2020/8883839

**Published:** 2020-11-27

**Authors:** Jong Youb Lim, Mi-Kyoung Oh, Jihong Park, Nam-Jong Paik

**Affiliations:** ^1^Department of Rehabilitation Medicine, Seoul National University College of Medicine, 103 Daehak-ro, Jongno-gu, Seoul 03080, Republic of Korea; ^2^Department of Rehabilitation Medicine, Daejeon Eulji University Hospital, 95, Dunsanseo-ro, Seo-gu, Daejeon 35233, Republic of Korea; ^3^Department of Rehabilitation Medicine, Seoul National University Bundang Hospital, 82, Gumi-ro 173 Beon-gil, Bundang-gu, Seongnam-si, Gyeonggi-do 13620, Republic of Korea

## Abstract

**Background:**

The prediction of motor recovery after stroke is an important issue, and various prediction models have been proposed using either clinical behavioral or neurological biomarkers. This study sought to identify the effects of clinical behavioral biomarkers combined with corticospinal tract (CST) injury measurement on the prediction of motor recovery after stroke.

**Methods:**

The region of interest was drawn on the normalized brain magnetic resonance imaging scans of patients with first-ever unilateral hemispheric stroke, and the degree of CST injury was calculated in a total of 67 such subjects. Patients who had initial minor deficits and showed a ceiling effect on motor recovery were excluded. To predict the follow-up Fugl-Meyer assessment (FMA) scores, correlation and regression analyses were performed using various clinical behavioral biomarkers, including age, sex, lesion location, and initial FMA scores and CST injury measurements.

**Results:**

Only the initial FMA-upper extremity (UE) score was statistically correlated with the follow-up FMA-UE score at ≥2 months after the onset (adjusted *R*^2^ = 0.626), and the relationship between CST injury and follow-up FMA-UE score was unclear (*n* = 53). Hierarchical clustering between the initial and follow-up FMA-UE scores showed three clusters. After exclusion of a cluster with an initial FMA-UE ≥ 35, the prediction of the follow-up FMA-UE score was possible by incorporating the initial FMA-UE score and CST injury measurements (*n* = 39). However, the explanatory power decreased (adjusted *R*^2^ = 0.445), and the unique contribution of the CST injury (10.1%) was lower than that of the initial FMA-UE score (26.7%). With respect to the FMA-lower extremity score, CST injury was not related to recovery.

**Conclusions:**

Motor recovery of the upper and lower extremities after stroke could be predicted using the initial FMA score. CST injury was significant for the prediction of motor recovery of the upper extremity in patients with severe initial motor deficits (FMA-UE < 35); however, its portion of prediction of motor recovery was low. The prediction of poststroke motor recovery using the initial motor deficit was not improved by the addition of CST injury measurements.

## 1. Introduction

Since motor impairment is the most common cause of disability in patients with stroke, the prediction of motor recovery after stroke has been and remains an important issue [1, 2]. The initial state of motor impairment of the upper extremity has been reported as the most influential predictor of upper extremity recovery [3]. However, the predictive ability of the initial severity alone is imperfect [4].

Early prediction of motor recovery is challenging, especially in patients with severe initial impairment [5], and the degree of corticospinal tract (CST) injury can be related to the motor outcomes of these patients [6]. The plateaued motor outcomes of patients in chronic stages of stroke are closely linked to the integrity of the CST, and a poor motor outcome is expected in cases of large CST involvement on magnetic resonance imaging (MRI) [7].

CST injury can be measured using various methods. Compared with clinical measures, assessing CST integrity according to the presence of motor-evoked potentials (MEPs) has been reported to have a better predictive ability for motor recovery [8]. Although MEPs have high specificity (99%), their sensitivity is remarkably variable (62–94%), as some patients whose initial MEPs were absent have been shown to partly recover their motor function [9]. Assessing CST integrity using diffusion tensor imaging (DTI) may reflect white matter degeneration by measuring the decrease in fractional anisotropy distal to the stroke area and by quantifying the involved number of CST fibers [8, 10]. However, DTI measurements along the CST in the first few weeks might not correctly reflect CST injury because Wallerian degeneration in the early stages is usually subtle and gradually develops thereafter. In addition, Wallerian degeneration is difficult to detect with this MRI technique [11].

Using conventional MRI is beneficial in terms of its availability and standardization [2]. To this end, spatial normalization has been carried out, in which lesion images are transformed into reference images of normal subjects [12, 13]. In studies in which the tract injury of each patient overlapped with the contralesional normal white tract, a strong correlation was found between the motor gain and the degree of injury of the tract from the motor cortex, whereas baseline motor function and infarct volume were weak predictors of treatment gains in patients with chronic stroke undergoing rehabilitation treatment [14]. Prediction models incorporating CST injury and clinical assessment were seen as being more precise than models using a single biomarker, whether a behavioral or neuroimaging-related one [15, 16].

Therefore, the purpose of this study was to determine the controversial effects of a combination of CST injury measurements and clinical behavioral biomarkers on the prediction of motor recovery after stroke.

## 2. Methods

### 2.1. Study Population

Patients who were initially admitted to the acute stroke unit and then transferred to the rehabilitation unit in the department of rehabilitation medicine of a tertiary university hospital for inpatient stroke neurorehabilitation from May 2003 to August 2017 were retrospectively reviewed. The inclusion criteria were (1) spontaneous first-ever ischemic or hemorrhagic stroke, (2) unilateral hemispheric lesion, (3) aged 18 years or older, and (4) complete Fugl-Meyer assessment (FMA) data, including at baseline and at follow-up after ≥2 months. The exclusion criteria were (1) traumatic causes, (2) concomitant lesions difficult to localize (subarachnoid hemorrhage, subdural/epidural hemorrhage, hydrocephalus, and Moyamoya disease), (3) nonhemispheric stroke (posterior circulation lesion), (4) bilateral lesion, (5) recurrent stroke, (6) incomplete medical records, (7) no brain MRI scan taken after the onset of stroke, (9) incomplete FMA data, (10) failed Diffeomorphic Anatomical Registration Through Exponentiated Lie algebra (DARTEL) normalization, and (11) complete CST involvement (100%), that is CST injury = 1.

Data on age, sex, lesion location, lesion side, and the date of stroke onset were also collected. The lesion location was classified as either cortical or subcortical, in which the area medial to the insular cortex and ventral to the genu of the corpus callosum was categorized as subcortical regions [17]. The study protocol was approved by the Institutional Review Board (IRB), and informed consent was waived by the IRB.

### 2.2. FMA Score Acquisition

FMA was evaluated after patients, who were initially admitted to the acute stroke unit, were transferred to the department of rehabilitation medicine. Patients with incomplete baseline FMA or follow-up FMA data were excluded. If there were two or more follow-ups for the FMA data, the FMA measured around 2 months poststroke was selected as the follow-up FMA.

### 2.3. Image Processing

The brain MRI scans of patients classified as having a spontaneous infarction or hemorrhage were assessed in a picture archiving and communication system (INFINITT PACS®, INFINITT Healthcare, Seoul, Korea) and downloaded in Digital Imaging and Communications in Medicine (DICOM®, National Electrical Manufacturers Association, Rosslyn, VA, USA) format. All included MRI scans were taken using a 3T MRI scanner (Achieva and Ingenia, Philips Healthcare, Best, Netherlands) with a SENSE head coil (Philips Healthcare). The DICOM® files were transformed into the Neuroimaging Informatics Technology Initiative (NIfTI) format by using MRIcron software (http://people.cas.sc.edu/rorden/mricron/index.html). To normalize the brain MRI scans to Montreal Neurological Institute template space, the DARTEL algorithm of SPM12 software (https://www.fil.ion.ucl.ac.uk/spm/software/spm12) based on MATLAB® version R2017b (MathWorks, Natick, MA, USA) was used. DARTEL-normalized images were manually inspected to check for image distortion on the left and right edges of the brain, the anterior and posterior ends of the corpus callosum, and the edges of the ventricles [18]. Images in which the normalization had failed were excluded.

### 2.4. Lesion Drawing

The region of interest (ROI) was drawn on the normalized image using MRIcron [19]. Referring to the original MRI, the ROI was traced on every slice that contained any lesion. The volume of interest (VOI) was saved in a NIfTI format. Lesion volume was measured by overlapping the VOI with the normalized image.

### 2.5. Quantification of CST Injury

The degree of CST injury was quantified according to the method reported by Riley et al. [14]. The CST was divided into 16 longitudinal subsections, and a subsection was considered injured when ≥5% of the subsection overlapped with the injury region. The percentage of CST injury was calculated by summing the injured subsections divided by the total number of subsections (i.e., 16). The right and left sides demonstrated values ranging from 0 to 1 (0–100%) [20]. Only patients with unilateral hemispheric lesions were included; subsequently, the nonlesion side showed a value of 0, and the value of the lesion side was used as reflective of the CST injury. Patients with a CST injury of 1 (100%) were excluded because recovery proportional to the CST injury in these patients could not be substantiated.

### 2.6. Upper and Lower Extremity Analyses

To prevent a ceiling effect, patients with an initial FMA of the hemiplegic upper extremity (FMA-UE) >59 were excluded in the upper extremity analysis. Because the total FMA-UE score is 66 and the minimal clinically important difference (MCID) for the FMA-UE is 6.6 points, a value of 59 was chosen [21]. The total FMA of the hemiplegic lower extremity (FMA-LE) is 34, and the MCID for the FMA-LE is 6 [22]; accordingly, patients with an initial FMA-LE > 27 were excluded to prevent a ceiling effect in the lower extremity analysis. Using the follow-up FMA score as the motor outcome, significant predictors were sought among multiple variables for the upper extremity and the lower extremity.

### 2.7. Statistical Analysis

Statistical analysis was performed using IBM SPSS® statistics version 19.0 (IBM Corp., Armonk, NY, USA). A value of *P* < 0.05 was considered statistically significant. A linear regression between CST injury and follow-up FMA score was performed. The follow-up FMA score and statistically significant variables in the univariate Pearson correlation analysis were entered in a multiple regression analysis. The presence of multicollinearity was assessed with a partial correlation analysis of the included variables while controlling for excluded variables.

Hierarchical clustering analysis between the initial and follow-up FMA scores was applied to discover whether specific patient groups [23], in whom CST injury was more helpful in the prediction of motor recovery, were present. If the correlation between the initial and follow-up FMA scores was strong [24], then principal component analysis was used for hierarchical clustering to avoid multicollinearity [25]. Agglomerative hierarchical clustering with Ward's method and squared Euclidean distance was used [26, 27], and the cutoff point of the clusters was established by the elbow method [28]. The relationship between these clusters and CST injury was assessed. If distinct clusters were discriminated with the initial FMA score, then the criterion was calculated using a receiver operating characteristic (ROC) curve analysis.

## 3. Results

### 3.1. Clinical Information

**Figure 1:**
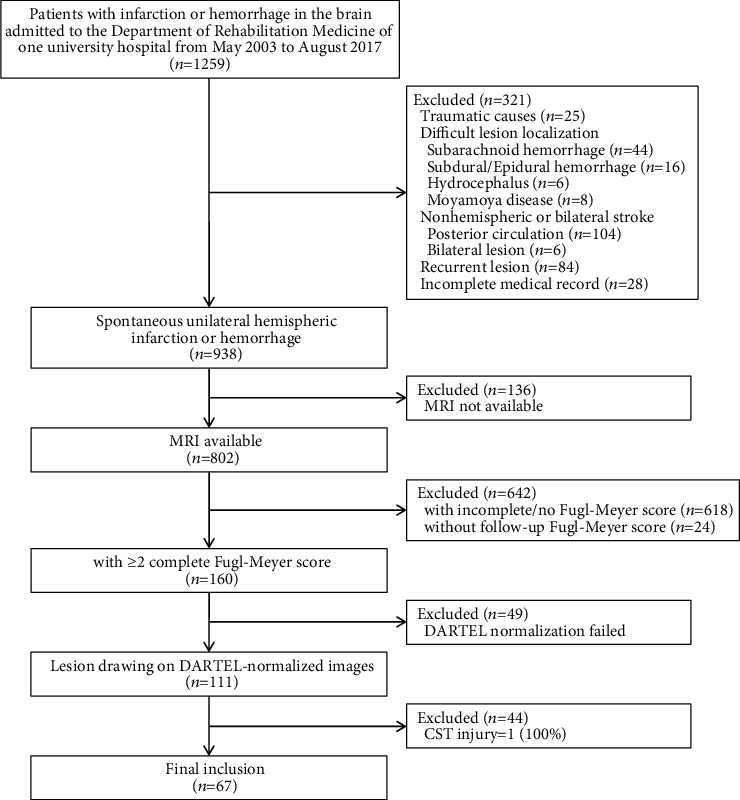
Flowchart of the study procedure. MRI: magnetic resonance imaging; DARTEL: Diffeomorphic Anatomical Registration Through Exponentiated Lie algebra; CST: corticospinal tract.

From the data of 1,259 patients, images, FMA, CST injury, and lesion volume were available from 67 patients (37 men and 30 women) after application of the exclusion criteria (Figure 1). The mean age was 67.82 ± 15.04 years. An MRI was performed 2.81 ± 6.47 days after stroke onset. The initial FMA evaluation was performed 11.37 ± 8.30 days after the onset. The follow-up FMA evaluation was performed 62.09 ± 85.02 days after stroke onset, and 53.66 ± 86.68 days had elapsed between the initial and follow-up FMA evaluations. Thirty-six patients had cortical lesions and 31 patients had subcortical lesions, and there were no patients with both cortical and subcortical lesions. Furthermore, 37 patients had right brain lesions and 30 patients had left brain lesions (Table 1).

### 3.2. Upper Extremity Analysis

**Figure 2:**
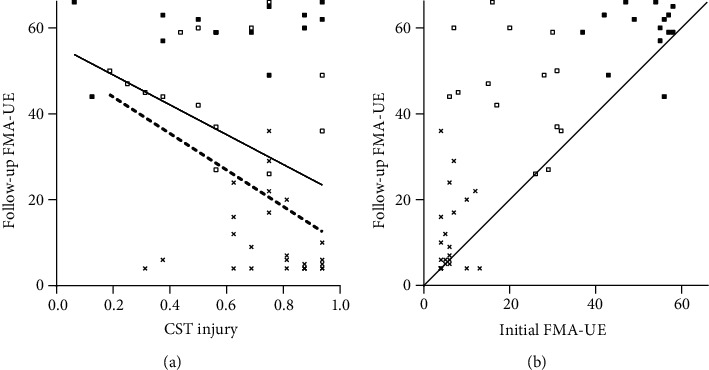
Follow-up Fugl-Meyer assessment of the hemiplegic upper extremity (FMA-UE). (a) Linear regression between corticospinal tract (CST) injury and follow-up FMA-UE scores. The symbols indicate the same patient clusters as those shown in (b). The solid line is the regression line, and the dashed line is the regression line after the exclusion of patients with an initial FMA-UE score of ≥35 (■). (b) Hierarchical clustering between initial and follow-up FMA-UE scores shows three clusters (■, □, and ×). The solid line is the line of identity.

After excluding patients with an initial FMA-UE score > 59 (*n* = 14), 53 patients were included in the upper extremity analysis. A linear regression between CST injury and follow-up FMA-UE score in these patients revealed the following values: *R*^2^ = 0.119, adjusted *R*^2^ = 0.102, *β* = −0.346, and *P* = 0.011 (Figure 2(a)). In a correlation analysis between the follow-up FMA-UE score and other variables, CST injury and initial FMA-UE score were found to be statistically significant (Table 2). A multiple regression analysis conducted between these two factors and follow-up FMA-UE score revealed that only the initial FMA-UE score was statistically significant. However, CST injury demonstrated no significant regression. A partial correlation analysis of CST injury and initial FMA-UE score controlling for excluded variables (age, sex, lesion location, and lesion volume) was not significant (*P* = 0.128); therefore, multicollinearity was not present between CST injury and initial FMA-UE score.

Because of the strong correlation between the initial and follow-up FMA-UE scores (Table 2), principal component analysis was used for hierarchical clustering between the initial and follow-up FMA-UE scores. The elbow method revealed three clusters (Figure 2(b)). When these groups were assessed in light of CST injury, these clusters were roughly maintained and patients with higher initial FMA-UE scores did not show a distribution that was proportional to CST injury (Figure 2(a)). The ROC curve of the initial FMA-UE score revealed this group as consisting of patients with an initial FMA-UE score ≥ 35.

After exclusion of this FMA-UE ≥ 35 group (*n* = 14), a linear regression between CST injury and follow-up FMA-UE in these patients (*n* = 39) revealed the following values: *R*^2^ = 0.208, adjusted *R*^2^ = 0.186, *β* = −0.456, and *P* = 0.004 (Figure 2(a)). In the correlation analysis between the follow-up FMA-UE score and other variables, CST injury and initial FMA-UE score were statistically significant (Table 2). In a multiple regression between these factors and follow-up FMA-UE score, both CST injury and initial FMA-UE score were statistically significant. Multicollinearity was not found in a partial correlation analysis of CST injury and initial FMA-UE score controlling for excluded variables (*P* = 0.187). The *R*^2^ value of this model was lower than that acquired with only the initial FMA-UE score before the exclusion of the patient cluster. Moreover, the unique contribution, which was calculated by squaring the semipartial correlation (Table 2), of the initial FMA-UE score was 26.7% and that of CST injury was 10.1%.

### 3.3. Lower Extremity Analysis

**Figure 3:**
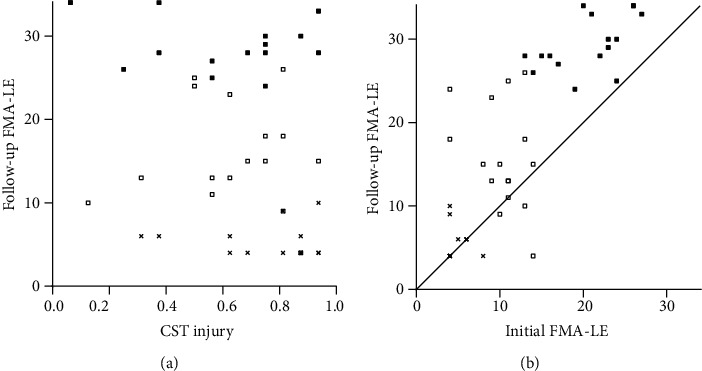
Follow-up Fugl-Meyer assessment of the hemiplegic lower extremity (FMA-LE). (a) Linear regression between corticospinal tract (CST) injury and follow-up FMA-LE scores. The symbols indicate the same patient clusters as those shown in (b). The regression is not found, even after the exclusion of patients with an initial FMA-LE score of ≥14 (■). (b) Hierarchical clustering between initial and follow-up FMA-LE scores shows three clusters (■, □, and ×). The solid line is the line of identity.

For the lower extremity analysis, 45 patients were included after excluding patients with an initial FMA-LE score > 27 (*n* = 22). A linear regression between CST injury and follow-up FMA-LE score in these patients did not show a statistical significance (*R*^2^ = 0.055, adjusted *R*^2^ = 0.033, *β* = −0.234, and *P* = 0.121) (Figure 3(a)). In a correlation analysis between the follow-up FMA-LE score and other variables, only the initial FMA-LE score was statistically significant (Table 3).

The correlation between the initial and follow-up FMA-LE scores was strong, and hierarchical clustering based on principal component analysis between the initial and follow-up FMA-LE scores showed three clusters by the elbow method (Figure 3(b)). When these groups were assessed in light of CST injury, these clusters were roughly maintained and patients with higher scores in both initial and follow-up FMA-LE scores did not show any distribution that was proportional to CST injury (Figure 3(a)). The ROC curve of the initial FMA-LE score identified this group as consisting of patients with an initial FMA-LE score ≥ 14.

Even after excluding this group (*n* = 17), a linear regression between CST injury and follow-up FMA-LE score in these patients (*n* = 28) was not significant (*R*^2^ = 0.015, adjusted *R*^2^ = −0.023, *β* = −0.122, and *P* = 0.536) (Figure 3(a)). A linear regression between initial FMA-LE and follow-up FMA-LE scores revealed a regressive trend, but the *R*^2^ value decreased more than that obtained before the hierarchical clustering-based exclusion (Table 3).

## 4. Discussion

In this study, the follow-up FMA score was predicted using the initial FMA score for the upper extremity and the lower extremity. CST injury showed correlation with upper extremity motor recovery only in patients with severe initial motor deficits, but the explanatory power of CST injury was low. Motor recovery of the upper extremity in patients with mild to moderate deficits and that of the lower extremity did not show any correlation with CST injury.

### 4.1. Upper Extremity Analysis

CST injury and initial FMA-UE score showed correlation with the follow-up FMA-UE score; however, this relationship was not noted in the multiple regression. Motor recovery after stroke has been reported to follow one of the following two patterns: (i) proportional recovery, in which approximately 70% of the maximal potential recovery is achieved, and (ii) nonproportional recovery, wherein limited or no recovery occurs [29]. Several recent studies argued that proportional recovery is merely a mathematical finding [30–32], whereas the degree of relationship between CST injury and motor impairment was considered to be moderate to high [16]. Poststroke upper limb recovery was reported to have various patterns of improvement, and five subgroups were identified in a recent study [33]. Therefore, we performed a cluster analysis not using *Δ*FMA-UE which was the main variable in the studies about proportional recovery, but using the initial and follow-up FMA-UE scores only to delineate the different patterns of recovery. With the elbow method, three clusters appropriate to the hierarchical clustering were found, and the one with a higher initial FMA-UE score showed a disproportionate relationship with CST injury. A multiple regression analysis, which was carried out after excluding this cluster, demonstrated a relationship between the initial and follow-up FMA-UE scores and CST injury without multicollinearity. In the excluded cluster, some ceiling effect was unavoidable because the improvement in FMA-UE scores was limited in patients with higher initial scores [30].

Our findings suggest that CST injury measured in early stages after stroke does not add significant predictive value to the long-term follow-up of plateaued upper extremity motor recovery than does the measurement of the initial FMA-UE score alone in all patients. The predictive value of CST injury was found only in patients with initial severe deficits with FMA-UE < 35, but the value was lower than that of the initial FMA-UE score. Moreover, the explanatory power of the prediction model made by combining CST injury and initial FMA-UE score in these patients was lower than that of only the initial FMA-UE score in all patients. This initial score (35) was different from 22, which was the reported value distinguishing patients with proportional recovery from those with limited recovery [2]. The same study also stated that a dichotomy based on the degree of CST injury could help distinguish patients with limited recovery from those with proportional recovery [2]. However, in our study, patients with high initial FMA-UE score showed high follow-up FMA-UE score, even with severe CST injury (Figure 2(a)). The correlation between CST injury and motor deficits at six months after the onset of stroke was found to be moderate, with *R* = 0.32–0.39 [34], comparable to the absolute value of the correlation coefficient *R* = −0.346 obtained in our study between CST injury and the follow-up FMA-UE score (Table 2).

### 4.2. Lower Extremity Analysis

CST injury did not show a correlation with the follow-up FMA-LE score, whereas the initial FMA-LE score showed a correlation. Even with the exclusion of one cluster that had an initial FMA-LE score ≥ 14, CST injury was still not related to the follow-up FMA-LE score, and the *R*^2^ values decreased in the regression analysis performed between the initial and follow-up FMA-LE scores.

This means that CST injury measured in early stages after stroke does not add significant predictive value to the long-term follow-up of plateaued lower extremity motor recovery compared to what the initial FMA-LE score measurement alone does. As in the upper extremity, the lower extremity was reported to show proportional recovery with [35] or without a subset of nonfitters [36]. Interestingly, the study, which presented a nonfitter group, suggested a FMA-LE score of 14 as a discriminant between fitters and nonfitters of proportional recovery [35].

### 4.3. Prediction of Motor Recovery

Motor recovery of the upper extremity could be predicted by the initial FMA-UE score. In patients with an initial FMA-UE score < 35, who had severe initial motor deficit, motor recovery could be predicted with the degree of CST injury and initial FMA-UE score; however, the predictive value was low and the contribution of CST injury was smaller than that of the initial FMA-UE score. Age, sex, lesion characteristics, and lesion volume were not found to be related in our study. Additionally, the *R*^2^ value in a multiple regression decreased after the exclusion of patients with a high initial FMA-UE score. Because the FMA-UE scores would have less chance to decrease than to increase [30], the majority of the follow-up FMA-UE scores were higher than or equal to the initial FMA-UE scores (Figure 2(b)). After exclusion of the patient cluster that showed a higher level of concentration than the other clusters, the relative distribution of the initial and follow-up FMA-UE scores had higher scattering, and a decreased *R*^2^ value was found in the linear regression performed between the initial and follow-up FMA-UE scores (Table 2). The excluded patients were detected as outliers in the relationship between CST injury and follow-up FMA-UE score (Figure 2(a)), and the removal of those outliers improved the *R*^2^ value of the correlation between CST injury and follow-up FMA-UE score. However, the decrease of the *R*^2^ value in the multiple regressions implies that the contribution of CST injury in the prediction of the follow-up FMA-UE score was smaller than that of the initial FMA-UE score. This was proven by the unique contribution, which was calculated by squaring the semipartial correlation (Table 2). The unique contribution of CST injury was 10.1% and that of the initial FMA-UE score was 26.7%.

To date, the best-known predictor of motor recovery is the initial motor impairment [3, 29], and its combined use with other neurological biomarkers may potentially improve the prediction [37–39]. The isolated or added value of CST integrity has been proven in previous studies [14, 40]. However, in the present study, the additive effect of CST injury measurements was not observed. A previous study using multiple dichotomies reported that no significant added value of MEPs, which assesses CST integrity, was found in the prediction of upper extremity motor recovery [41]. A recent review dealing with the predictors of poststroke motor recovery stated that there was still no consensus regarding the method of assessing CST integrity [42]. It is presumed that the contribution of CST integrity was not high in the prediction of motor recovery, and a complex prediction model incorporating categorization and multiple regressions might be appropriate to predict poststroke motor recovery more accurately.

The small contribution of CST integrity in the upper extremity motor recovery was reflected in the PREP2 algorithm, which uses MEPs only, in a subset of patients [43]. The PREP2 algorithm uses shoulder abduction and finger extension as the first discriminators [44], and patients with an initial low function are classified using MEPs measured at 5–7 days after stroke [43]. However, when the CST injury is small, the recovery of MEPs is possible even in cases without initial MEPs [45]. Of patients with poor initial function, those with smaller MEPs rather than larger MEPs showed more motor improvement with robotic movement therapy [32]. Furthermore, some patients with the presence of MEPs in the early stage after stroke did not recover [29]. Therefore, prediction with MEPs should be interpreted with caution. There might be a potential of using CST injury measurements for the prediction of the motor recovery only in patients with severe initial motor impairment.

With regard to the lower extremity, the initial FMA-LE score could predict the follow-up FMA-LE score. CST injury showed no correlation or regression with the follow-up FMA-LE score, regardless of patients' initial severity. Recovery of the lower extremity was known to be less related to CST than the recovery of the upper extremity [46]. The role of the extrapyramidal tracts, including the corticoreticulospinal tract, was one of the proposed mechanisms for the recovery of the lower extremity [47, 48].

Neuroanatomically, some aspects of motor recovery after stroke could not be explained by ipsilesional CST injury alone, and the CST descending from the contralesional hemisphere was suggested to play a role in recovery [34]. During recovery from hemiplegia, the activation of both ipsilesional and contralesional areas occurs [29]. This phenomenon may be one reason for which the recovery was unrelated to ipsilesional CST.

This study has several limitations. First, although a large number of patients were initially enrolled, a relatively small number of patients were finally included. This was mainly due to the missing values of follow-up FMA scores. DARTEL normalization was another reason for excluding patients. Although the DARTEL algorithm is a reliable method for normalization [49], shrinkage of about 10% of the lesion volume was reported [50]. Prospective studies on acute stroke patients reported low recruitment rates (0.26–0.41 participants/site/month) [34], and the number of included patients reviewed in this study was within this rate. Multiple steps for the inclusion [51], recurrence of a stroke [52], and no paresis at the time of recruitment [53] were among the suggested reasons for patient exclusion. This study had multiple stages for the selection of patients (Figure 1), and patients with recurrent stroke and those with high initial FMA scores were also excluded. Multicenter trials can be an option to overcome this high exclusion rate. Second, the time interval between MRI and initial FMA acquisition was more than a week. The patients underwent MRI at an early stage for the diagnosis of stroke at the time of the hospital visit. The FMA was measured after the patient was transferred to the department of rehabilitation medicine. This time interval was inevitable because this study has a retrospective design; however, the condition and cooperation of the patient during acute stroke care could be inconsistent and unstable, and therefore, an initial FMA may be evaluated in the department of rehabilitation medicine. Nonetheless, some patients are likely to have had a low initial function, which improved greatly within a week, and they would have been excluded as patients with near-full function to prevent a ceiling effect. Further prospective studies with a strict schedule of measurement will be needed. Third, because this study was retrospective in nature, possible covariates were not controlled for. Age and sex were entered into the analysis as respective variables; however, cognitive function, comorbidity, and additional symptoms (e.g., aphasia, hemispatial neglect, and ataxia) that could affect the motor outcomes were not considered. Fourth, although the anatomical CST injury measurements used in this study have been assessed in several studies in the prediction of motor recovery [14, 54], the addition of neurophysiological assessments, such as the assessment of the presence of MEPs, may make the prediction more robust [40]. Lastly, anatomical measurements of CST injury in the early stages of stroke may not accurately reflect later stage motor recovery, and measurements in later stages after Wallerian degeneration may more accurately reflect the motor outcomes because Wallerian degeneration is a lengthy process [11].

## 5. Conclusions

Motor recovery of the upper and lower extremities after stroke could be predicted by the initial FMA scores. CST injury was correlated with upper extremity motor recovery only in patients with initial FMA-UE < 35; however, the explanatory power of the combination of CST injury and initial FMA-UE score in these patients was lower than that of the initial FMA-UE score alone in all patients, and the unique contribution of CST injury was lower than that of the initial FMA-UE score. The prediction of poststroke upper extremity motor recovery by the initial FMA-UE score was not improved with the addition of CST injury measurements.

## Figures and Tables

**Table 1:** Demographic and clinical characteristics of the patients.

Patient characteristics	*n* = 67
Sex, Male/Female	37/30
Age, years	67.82 ± 15.04
Lesion of stroke, Cortical/Subcortical	36/31
Side of stroke, Right/Left	37/30
Time from stroke	
MRI, days	2.81 ± 6.47
Initial FMA, days	11.37 ± 8.30
Follow-up FMA, days	62.09 ± 85.02
Days between FMAs	53.66 ± 86.68

MRI: magnetic resonance imaging; FMA: Fugl-Meyer assessment.

**Table 2:** Correlation and multiple regression analyses between FMA-UE and other variables.

Correlation	Total (*n* = 53)				Initial FMA-UE <35 (*n* = 39)			
	Correlation coefficient			*P* value	Correlation coefficient			*P* value
Age	−0.015			0.917	−0.076			0.646
Sex	−0.044			0.753	0.130			0.430
Lesion location	0.073			0.603	0.089			0.591
CST injury	−0.346			0.011^∗^	−0.456			0.004^∗^
Lesion volume	−0.098			0.487	−0.045			0.787
Initial FMA-UE	0.789			<0.001^∗^	0.611			<0.001^∗^
Multiple regression	*β*	*P* value	Partial^†^	Part^‡^	*β*	*P* value	Partial^†^	Part^‡^
Initial FMA-UE	0.750	<0.001^∗^	0.769	0.722	0.532	<0.001^∗^	0.580	0.517
CST injury	−0.141	0.117	−0.220	−0.135	−0.328	0.012^∗^	−0.402	−0.318
	*R^2^* = 0.641, adjusted *R^2^* = 0.626				*R^2^* = 0.475, adjusted *R^2^* = 0.445			

FMA-UE: Fugl-Meyer assessment of the hemiplegic upper extremity; CST: corticospinal tract. ^∗^*P* <0.05. ^†^Partial correlations (shared contributions) of the variables. ^‡^Semipartial correlations (unique contributions) of the variables.

**Table 3:** Correlation and regression analyses between FMA-LE and other variables.

Correlation	Total (*n* = 45)		Initial FMA-LE <14 (*n* = 28)	
	Correlation coefficient	*P* value	Correlation coefficient	*P* value
Age	−0.169	0.267	−0.226	0.247
Sex	−0.044	0.772	0.106	0.593
Lesion location	−0.028	0.857	0.096	0.626
CST injury	−0.234	0.121	−0.122	0.536
Lesion volume	−0.192	0.206	−0.069	0.725
Initial FMA-LE	0.824	<0.001^∗^	0.580	0.001^∗^
Linear regression	*β*	*P* value	*β*	*P* value
Initial FMA-LE	0.824	<0.001^∗^	0.580	0.001^∗^
	*R^2^* = 0.680, adjusted *R^2^* = 0.672		*R^2^* = 0.336, adjusted *R^2^* = 0.311	

FMA-LE: Fugl-Meyer assessment of the hemiplegic lower extremity; CST: corticospinal tract. ^∗^*P* <0.05.

## Data Availability

The data used to support the findings of this study are restricted by the IRB of Seoul National University Bundang Hospital in order to protect patient privacy. Data are available from the corresponding author Prof. Nam-Jong Paik (njpaik@snu.ac.kr) for researchers who meet the criteria for access to confidential data.
